# Prevalence of malignant hyperthermia diagnosis in obstetric patients in the United States, 2003 to 2014

**DOI:** 10.1186/s12871-020-0934-0

**Published:** 2020-01-20

**Authors:** Jean Guglielminotti, Henry Rosenberg, Guohua Li

**Affiliations:** 1 Department of Anesthesiology, Columbia University College of Physicians and Surgeons, 622 West 168th Street, PH5-505, New York, NY 10032 USA; 2 the Malignant Hyperthermia Association of the United States, Sherburne, NY 13460 USA; 3 Department of Epidemiology, Columbia University Mailman School of Public Health, 722 West 168th Street, New York, NY 10032 USA

**Keywords:** Childbirth, Malignant hyperthermia, Obstetrics

## Abstract

**Background:**

The cost-benefit of stocking dantrolene in maternity units for treating malignant hyperthermia (MH) has been recently questioned because of the low incidence of MH crisis in the general population and the low utilization of general anesthesia in obstetrics. However, no study has examined the prevalence of MH susceptibility in obstetrics. This study aimed to assess the prevalence of MH diagnosis and associated factors in obstetric patients.

**Methods:**

Data for this study came from the National Inpatient Sample from 2003 to 2014, a 20% nationally representative sample of discharge records from community hospitals. A diagnosis of MH due to anesthesia was identified using the *International Classification of Diseases, Ninth Revision, Clinical Modification* code 995.86. MH prevalence was estimated according to the delivery mode and patient and hospital characteristics.

**Results:**

During the 12-year study period, 47,178,322 delivery-related discharges [including 15,175,127 (32.2%) cesarean deliveries] were identified. Of them, 215 recorded a diagnosis of MH, yielding a prevalence of 0.46 per 100,000 [95% confidence interval (CI), 0.40 to 0.52]. The prevalence of MH diagnosis in cesarean deliveries was 0.81 per 100,000 (95% CI, 0.67 to 0.97), compared with 0.29 per 100,000 (95% CI, 0.23 to 0.35) in vaginal deliveries (*P* <  0.001). Multivariable logistic regression revealed that cesarean delivery was associated with a significantly increased risk of MH diagnosis [adjusted rate ratio (aOR) 2.88; 95% CI, 2.19 to 3.80]. Prevalence of MH diagnosis was lower in Hispanics than in non-Hispanic whites (aOR 0.47; 95% CI, 0.29 to 0.76) and higher in the South than in the Northeast census regions (aOR 2.44; 95% CI, 1.50 to 3.96).

**Conclusion:**

The prevalence of MH-susceptibility is about 1 in 125,000 in cesarean deliveries, similar to the prevalence reported in non-obstetrical surgery inpatients. The findings of this study suggest that stocking dantrolene in maternity units is justified.

## Background

Malignant hyperthermia (MH) is a pharmacogenetic disorder of the skeletal muscle triggered by halogenated inhalational agents or the depolarizing muscle relaxant succinylcholine [1, 2]. Exposure to triggering agents can lead to a rapid and uncontrolled calcium release in skeletal muscle cells cytoplasm and to a potentially lethal hyper-metabolic crisis among susceptible patients [3]. Exposure to succinylcholine only, without volatile anesthetics, can also triggers a MH crisis [3, 4]. MH susceptibility is related to defects in the ryanodine receptor 1 (RyR1) or in the Ca_v_1.1 channel, two calcium channels located in the sarcoplasmic reticulum membrane, or in the Stac3 protein required for effective colocation of RyR1 and Ca_v_1.1 [5]. Defects are caused by mutations in the genes encoding for RyR1, Ca_v_1.1 (CACNA1S), or Stac3. Despite the increased awareness of this condition, improved intraoperative monitoring of end-tidal carbon dioxide allowing early detection of MH crisis, and availability of an effective treatment (dantrolene), prognosis of MH crisis is still poor. The 2007–2012 report from the North American Malignant Hyperthermia Registry of the Malignant Hyperthermia Association of the United States indicates a case fatality rate of nearly 10%, with increases in MH-associated morbidity and mortality when dantrolene administration is delayed [3, 6, 7].

MH susceptibility is a rare disorder. Reported prevalence of MH diagnosis in studies using hospital discharge records from administrative data ranges from 0.18 per 100,000 (95% confidence interval (CI), 0.12–0.25) in ambulatory surgery center patients to 0.96 (95% CI, 0.75–1.41) in surgical inpatients [8, 9]. The low frequency of MH crises and the cost of stocking dantrolene have given rise to concern about the cost-benefit of the recommendation of the Malignant Hyperthermia Association of the United States (MHAUS) that dantrolene be made immediately available (for administration within 10 min) in operating room areas, especially in facilities with a low utilization of general anesthesia and triggering agents such as maternity units [10, 11]. MHAUS is a patient safety and advocacy organization and its recommendations are used by accrediting agencies, such as the Joint Commission, to assess preparedness for an MH event during survey visits. However, no study has specifically examined the prevalence of MH susceptibility in obstetric patients [12].

Using a nationally representative sample of community hospital discharges records in the United States between 2003 and 2014, we estimated the prevalence of MH diagnosis in delivery-related discharges and assessed patient- and hospital-level factors associated with the prevalence of MH diagnosis.

## Methods

### Data system and study sample

Data for this study came from the National Inpatient Sample (NIS). NIS is part of the Healthcare Cost and Utilization Project (HCUP) sponsored by the Agency for Healthcare Research and Quality. It is a stratified sample of approximately 20% of discharge records from community hospitals in the United States, excluding rehabilitation and long-term acute care hospitals. Community hospitals are defined as “all non-Federal, short-term, general, and other specialty hospitals, excluding hospital units of institutions.” Included among community hospitals are specialty hospitals such as obstetrics-gynecology, ear-nose-throat, orthopedic, and pediatric institutions. Also included are public hospitals and academic medical centers. The sampling procedure is stratified according to 6 hospital characteristics: census geographical area, urban or rural location, ownership, teaching status, and number of beds.

For each discharge, the NIS includes patient characteristics, and up to 15 procedural codes and 30 diagnostic codes defined in the *International Classification of Diseases, Ninth Revision, Clinical Modification* (ICD-9-CM). Discharges are weighted to permit inferences for a nationally representative population. Sampling weights before 2011 were updated in 2012 to take into consideration the 2012 sampling re-design. Detailed information on NIS data, methodology, and variables is publicly available (http://www.hcup-us.ahrq.gov/nisoverview.jsp).

The study sample consisted of all discharges indicating labor and delivery in the NIS between January 1, 2003, and December 31, 2014. They were identified with a combination of ICD-9-CM diagnosis and procedure codes developed by Kuklina et al. [13]

### Outcome

The outcome was a diagnosis of malignant hyperthermia (MH) recorded in the discharge record, identified according to the ICD-9-CM diagnosis code 995.86 (“Malignant hyperthermia due to anesthesia”).

### Patient and hospital characteristics

Patient age, race or ethnicity, hospital length-of-stay, and vital status at discharge were recorded directly from the NIS. In the NIS, Hispanic ethnicity is considered as a distinct racial/ethnic group. Excess hospital stay was defined as a stay greater than the 90th percentile (3 days for a vaginal delivery and 5 days for a cesarean delivery). The Charlson comorbidity index and the comorbidity index for obstetric patients were calculated using previously described ICD-9-CM algorithms [14–16]. The comorbidity index for obstetric patients was designed specifically for use in obstetric patient populations. It includes maternal age and 20 maternal conditions (e.g., severe preeclampsia/eclampsia), that are predictive of maternal end-organ injury or death during the delivery hospitalization through 30 days postpartum. Delivery was categorized as vaginal or cesarean delivery. The following hospital characteristics were recorded directly from the NIS: location and teaching status (categorized as rural, urban non-teaching and urban teaching) and geographic region defined by the US Census Bureau (Northeast, Midwest, South, and West).

### Statistical analysis

Statistical analysis was performed with R version 3.5.0 (R Foundation for Statistical Computing, Vienna, Austria) and specific packages (survey for analysis of complex survey samples). It followed the HCUP recommendations for the analysis of complex survey data [17].

Results are expressed as count (% or per 100,000). Both unweighted and weighted counts were calculated using individual discharge weight provided by the NIS. Univariate comparison of categorical variables between discharges with and without the diagnosis of MH used Chi-squared test and of continuous variables Wilcoxon tests.

The prevalence of MH diagnosis was calculated overall and according to the delivery mode (cesarean or vaginal delivery). Furthermore, stratified analysis was performed according to: 1) patient age, race, and comorbidity indexes; 2) hospital location/teaching status and geographic region; and 3) 3 time periods (2003–2006, 2007–2010, and 2011–2014).

The adjusted odds ratios (aOR) of MH diagnosis were estimated using a weighted multivariable logistic regression model with MH as the independent variable, the individual discharge weight provided by the NIS as weight, and the following variables as dependent variables: age, race, Charlson comorbidity index, comorbidity index for obstetric patients, mode of delivery (vaginal or cesarean), hospital location and teaching status, geographic region, and the 3 time periods. For categorical variables with missing values, a dummy indicator for missing values was created.

## Results

**Fig. 1:**
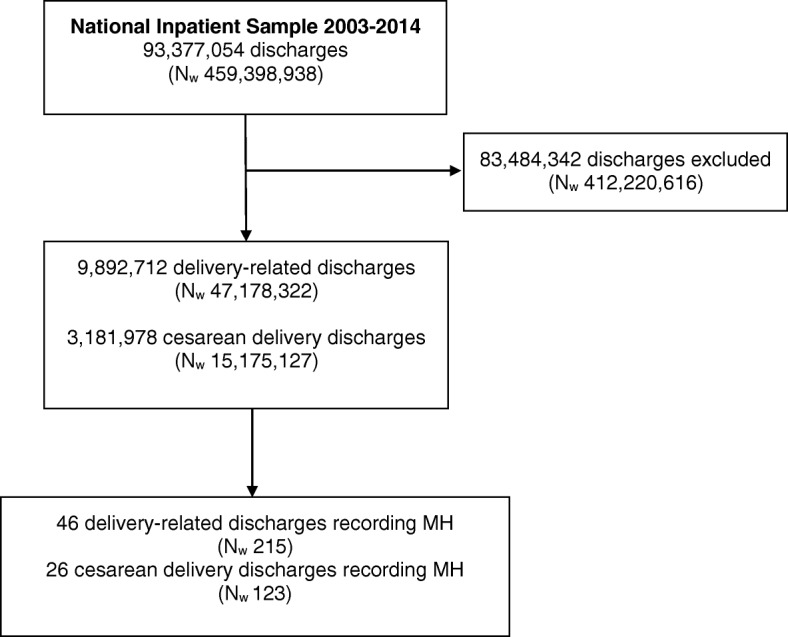
Flowchart of the study (MH: malignant hyperthermia; N_w_: weighted number)

During the 12-year study period, 9,892,712 delivery-related discharges were identified in the NIS (weighted number 47,178,322) (Fig. 1). Of them, 3,181,978 (32.2%) were cesarean delivery discharges (weighted number 15,175,127).

**Fig. 2:**
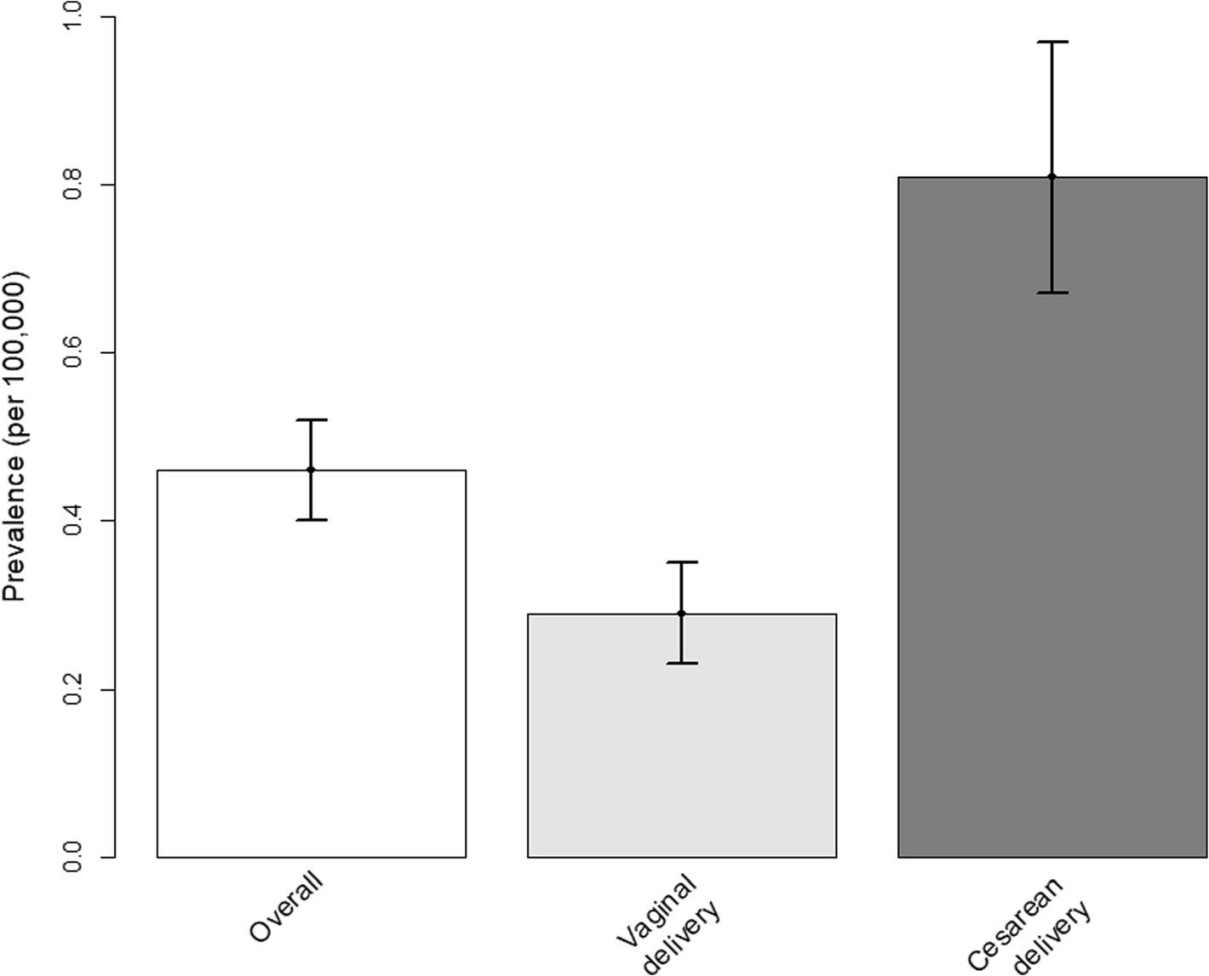
Prevalence of malignant hyperthermia diagnosis in obstetrics in the 47,178,322 delivery-related discharges of the 2003–2014 National Inpatient Sample, overall and according to the delivery mode. The vertical bars indicate the 95% confidence intervals

A diagnosis of MH was recorded in 46 delivery-related discharges (weighted number 215), yielding a prevalence of MH in obstetrics of 0.46 per 100,000 delivery-related discharges (95% confidence interval (CI), 0.40–0.52) (Fig. 2). In cesarean delivery discharges, a diagnosis of MH was recorded in 26 cases (weighted number 123), yielding a prevalence of 0.81 per 100,000 (95% CI, 0.67–0.97). In vaginal delivery discharges, a diagnosis of MH was recorded in 20 cases (weighted number 92), yielding a prevalence of 0.29 per 100,000 (95% CI, 0.23–0.35). The prevalence of MH in cesarean delivery discharges was significantly higher than in vaginal delivery discharges [*P*-value < 0.001; crude OR 2.83; 95% CI, 2.16 to 3.71; aOR 2.88; 95% CI, 2.19 to 3.80].

Prevalence of MH in delivery-related discharges according to patient characteristics, hospital characteristics, and time period is presented in Table 1. On the bivariate level, the prevalence of MH diagnosis was significantly lower in Hispanics than in non-Hispanic Whites and significantly higher in the Midwest and South census regions than in the Northeast census region, and in rural hospitals than in urban hospitals. No significant difference was observed across age groups, comorbidity indexes groups, or time periods. Multivariable adjustment revealed two factors associated with MH diagnosis: race/ethnicity (Hispanic versus non-Hispanic White, aOR 0.47; 95% CI, 0.29 to 0.76) and geographic census region (South versus Northeast, aOR 2.44; 95% CI, 1.50 to 3.96).

**Table 1:** Prevalence of malignant hyperthermia (MH) according to selected characteristics in the 47,178,322 delivery-related discharges of the 2003–2014 National Inpatient Sample

	Discharges (N_w_)	Discharges with MH (N_w_)	Prevalence (per 100,000; 95% CI)	*P*-value	Crude OR (95% CI)	Adjusted OR ^a^ (95% CI)
Patient characteristic						
Age (year)				0.67		
≤ 19	4,399,524	18	0.41 (0.24–0.65)		0.96 (0.58–1.58)	1.02 (0.62–1.67)
20–29	24,436,487	106	0.43 (0.36–0.52)		Reference	Reference
30–39	17,029,672	86	0.51 (0.40–0.62)		1.17 (0.88–1.56)	1.15 (0.86–1.54)
≥ 40	1,310,043	--^b^	--^b^ (0.12–0.89)		--^b^ (0.33–2.11)	0.85 (0.33–2.16)
Race				0.006		
White, Non-Hispanic	20,333,243	102	0.50 (0.41–0.61)		Reference	Reference
Black	5,268,451	24	0.46 (0.29–0.68)		0.90 (0.58–1.40)	0.82 (0.52–1.29)
Hispanic	9,035,771	20	0.22 (0.14–0.34)		0.44 (0.27–0.71)	0.47 (0.29–0.76)
Other	4,166,949	15	0.36 (0.20–0.59)		0.70 (0.40–1.20)	0.78 (0.45–1.35)
Charlson comorbidity index				0.85		
0	47,035,792	215	0.46 (0.40–0.52)		–	–
≥ 1	142,530	0	0.00 (0.00–2.59)		–	–
Patient						
Comorbidity index for obstetric patients				0.92		
0 or 1	41,467,932	188	0.45 (0.39–0.52)		Reference	Reference
≥ 2	5,707,789	27	0.47 (0.31–0.69)		1.06 (0.71–1.58)	0.79 (0.52–1.19)
Hospital						
Location and teaching status				0.023		
Rural	5,270,124	37	0.70 (0.49–0.97)		1.63 (1.12–2.39)	1.34 (0.91–1.98)
Urban non-teaching	19,230,712	83	0.43 (0.34–0.54)		1.01 (0.75–1.36)	0.96 (0.71–1.30)
Urban teaching	22,458,351	96	0.43 (0.35–0.52)		Reference	Reference
Hospital						
Census region				< 0.001		
Northeast	7,678,323	20	0.26 (0.16–0.40)		Reference	Reference
Midwest	10,088,837	51	0.51 (0.38–0.66)		1.96 (1.17–3.29)	1.71 (0.99–2.93)
South	17,827,228	114	0.64 (0.53–0.77)		2.47 (1.54–3.98)	2.44 (1.50–3.96)
West	11,583,934	29	0.25 (0.17–0.36)		0.98 (0.55–1.73)	1.11 (0.62–1.98)
Time period				0.17		
Year						
2003–2006	16,163,993	86	0.53 (0.43–0.66)		Reference	Reference
2007–2010	16,049,577	69	0.43 (0.33–0.54)		0.81 (0.59–1.11)	0.81 (0.59–1.12)
2011–2014	14,964,752	59	0.39 (0.30–0.51)		0.75 (0.54–1.04)	0.77 (0.55–1.09)

Abbreviation: *CI* confidence interval, *N*_*w*_ weighted number, *OR* odds ratio

^a^ Adjustment used all the variables listed in this table, along with the mode of delivery (vaginal or cesarean)

^b^ Because of HCUP data use agreement restrictions on small cell size, the number of observed cases and exact proportions are not presented

All the patients with a diagnosis of MH were alive at the time of discharge. The proportion of discharges with an excess length of hospital stay (i.e., 3 days for a vaginal delivery and 5 days for a cesarean delivery) was higher in discharges with a diagnosis of MH than in discharges without a diagnosis of MH (15.3% versus 4.1%, *P*-value < 0.001).

## Discussion

This study was designed to help close a knowledge gap in the epidemiology of MH. Specifically, we provide an estimate of the prevalence of MH diagnosis in the obstetric patient population using a nationally representative sample over a 12-year period. The prevalence of 0.81 per 100,000 (95% CI, 0.67 to 0.97) in cesarean delivery discharges is similar to the one reported in non-obstetrical surgery inpatients. Using New York State administrative data from 2001 to 2005, Brady et al. reported a prevalence of MH diagnosis in inpatient surgical discharges of 0.96 (95% CI, 0.67 to 1.24) [8]. The prevalence reported in the present study is lower than the rate previously reported using NIS data from 2000 to 2005 for the general inpatient population (1.12 per 100,000, 95% CI, 1.08 to 1.17) [18]. The discrepancy is likely due to the difference by the study populations as our analysis is restricted to obstetric patients and the prevalence of MH diagnosis in women is only about a third of the one in men [8, 19].

We identified several patient- and hospital-level characteristics associated with a higher prevalence of MH diagnosis. We observed a lower prevalence of MH diagnosis in Hispanics compared with non-Hispanic White patients. To our knowledge, this finding has not been reported before and if confirmed, warrants further investigation to elicit the underlying mechanisms. The lower prevalence of MH diagnosis in Hispanic obstetric patients is unlikely due to a lower utilization of general anesthesia and exposure to triggering agents because two previous studies have reported a higher utilization of general anesthesia in minority patients, including Hispanics [20, 21]. We also observed a geographic variation in the prevalence of MH diagnosis, with an elevated prevalence in the South region. Geographical variation is explained by the concentration of MH families in a given geographic area [22]. Rosero et al. also observed a higher prevalence of MH diagnosis in the South census region. Last, Rosero et al. reported an increase in the prevalence in MH diagnosis between 2001 and 2005 [18]. We do not confirm this trend over the longer 2003–2014 time period. The study by Rosero started just 2 years after the introduction of the ICD-9-CM code for MH in 1998 and the increase in this study may merely be related to the novelty of the code.

The prognosis of MH crisis remains poor with a fatality rate of nearly 10% and MH-associated morbidity and mortality increased when dantrolene administration is delayed [6, 7]. Similar poor prognosis is observed in studies using administrative data. In these studies, discharges recording a diagnosis of MH have a fatality rate ranging from 12 to 22% [8, 18]. In our study, we identified no death in discharges with a diagnosis of MH. A possible explanation for this finding is the young age of the patients analyzed and the lack of associated comorbidity; 98% of MH discharges were under 40 and only 0.3% had a Charlson comorbidity index greater than 1. By contrast, Brady et al. reported a 22% fatality rate in discharges with a diagnosis of MH but 49% of the MH discharges were older than 45 and 46% had a Charlson comorbidity index greater than 1 [8].

The Malignant Hyperthermia Association of the United States recommends that “*dantrolene must be available for all anesthetizing locations where MH trigger agents are used.*” This recommendation also applies to facilities where volatile agents are not available or administered, and succinylcholine is only stocked on site for emergency purposes [3]. The cost of an MH cart in maternity unit for responding to MH crisis has been estimated at about $2000 per year, including cost for the dantrolene itself, cost for the cart and non-dantrolene items, and cost for MH cart maintenance [10]. Ho et al. have recently questioned this recommendation based on cost-benefit analysis of stocking dantrolene in maternity units [10]. The low incidence of MH crisis and the low utilization of general anesthesia and triggering agents in obstetrics would not make stocking dantrolene policy cost-beneficial. Similar concerns have also been raised in ambulatory surgery centers but two recent studies support the dantrolene stocking policy in these centers [3, 23]. Of note, the prevalence of MH susceptibility of 0.81 per 100,000 cesarean deliveries (or 1 in 123,456 cases) reported in our study, similar to the prevalence reported in non-obstetrical surgery inpatients, suggests that stocking dantrolene in maternity units is justified. In our opinion, the low annual cost of a MH cart to treat a MH crisis in a maternity unit ($2000 per year) and the prevention of maternal death from a potentially treatable complication provide adequate justifications for stocking dantrolene in maternity units. This is particularly relevant in the current US context of implementing nationwide initiatives to reduce preventable severe maternal morbidity and mortality [24].

It is worth noting the limitations with our study. First, the NIS is a stratified sample of hospital discharges records and our study sample includes only 20% of childbirths during the study period. Second, identification of MH diagnosis relies on the accuracy of coding in discharges data and is susceptible to misclassification because of error or absence of coding. In a previous study evaluating the accuracy of MH diagnosis in hospital discharge records, we found that the ICD-9-CM code 995.86 (“Malignant hyperthermia due to anesthesia”) corresponded to a MH crisis in 24% of the cases, to a MH history in 47%, and to fever unrelated to MH in 24% [25]. Therefore, most of the cases with an MH diagnosis identified in the present study should be regarded as MH susceptibility rather than incident MH episodes. Lack of information on anesthetics exposed or dantrolene administration in the NIS dataset precludes us from identifying incident MH episodes. In other words, an ICD-9-CM-based approach favors sensitivity over specificity. Clinical registries such as the North American Malignant Hyperthermia Registry represent more specific approaches but also suffer from limitations such as the voluntary declarations of MH crisis with a risk of under declaration and underestimation of the prevalence of MH crisis. However, these two approaches should not be viewed as antagonistic but as complementary. Third, our unit of analysis was the discharge record and not the patient. So, a patient with multiple hospitalizations during this 12-year study could be included more than once in our analysis. However, the National Inpatient Sample is a 20% sample of discharges records, which decreases the likelihood of a patient being sampled and included multiple times. To further assess the potential impact of repeated admissions of MH-susceptible patients, we checked individually linked hospital discharge records for 2,851,697 obstetric patients in New York State from 2003 to 2014; of the discharges with a diagnosis of MH, none involved repeated admissions. Fourth, this study was limited to obstetric patients because of the recent controversy surrounding dantrolene stocking policy in maternity units. There are other settings where triggering agents may be used, especially succinylcholine, such as emergency departments or intensive care units and where stocking dantrolene could be suggested. To date, no study has specifically examined the prevalence of MH diagnosis in these locations. Fifth, a diagnosis of MH in women who underwent vaginal delivery may appear surprising because a general anesthetic with exposure to MH triggering agents is not required for an uncomplicated vaginal delivery. However, a general anesthetic may be required after a vaginal delivery in some circumstances such as the removal of a retained placenta or an exploration of the uterine cavity because of a postpartum hemorrhage. Moreover, as indicated in our previous study, most of the MH diagnoses in hospital discharge records refer to positive MH history and susceptibility rather than acute MH episodes [25]. These two factors may explain why the prevalence of MH diagnosis in vaginal delivery was much lower than in cesarean delivery (0.29 per 100,000 versus 0.81 per 100,000. Finally, the NIS does not capture all childbirths occurring in the country. For example, in 2014, the NIS included 3,828,643 childbirths which corresponds to 96% of the 3,988,076 births reported by the US National Center for Health Statistics. The “missing” 4% can be partially explained by out-of-hospital births (births at home or in a birth center), which accounted for 1.5% of live births in 2014.

## Conclusions

The prevalence of MH-susceptibility is about 1 in 125,000 in cesarean deliveries, similar to the prevalence reported in non-obstetrical surgery inpatients. Results of this study suggest that stocking dantrolene in maternity units is justified.

## Data Availability

The data that support the findings of this study are available from the Healthcare Cost and Utilization Project (HCUP) (https://www.hcup-us.ahrq.gov/) but restrictions apply to the availability of these data, which were used under the HCUP data user agreement for the current study, and so are not publicly available.

## Abbreviations

aOR: Adjusted Odds Ratio
CI: Confidence Interval
HCUP: Healthcare Cost and Utilization Project
ICD-9-CM: International Classification of Diseases, Ninth Revision, Clinical Modification
MH: Malignant Hyperthermia
MHAUS: Malignant Hyperthermia Association of the United States
NIS: National Inpatient Sample
OR: Odds Ratio
US: United States
